# Mesenchymal Stem Cell‐Based Therapy for Cerebellar Ataxia: From Bench to Bedside

**DOI:** 10.1002/cns.71038

**Published:** 2026-07-23

**Authors:** Kyoungho Suk, Ho‐Won Lee, Sang Ryong Kim

**Affiliations:** ^1^ Department of Pharmacology, School of Medicine Kyungpook National University Daegu Republic of Korea; ^2^ Brain Science & Engineering Institute Kyungpook National University Daegu Republic of Korea; ^3^ BK21 FOUR KNU Convergence Educational Program of Biomedical Sciences for Creative Future Talents Kyungpook National University Daegu Republic of Korea; ^4^ Department of Neurology Kyungpook National University Chilgok Hospital Daegu Republic of Korea; ^5^ School of Life Sciences Kyungpook National University Daegu Republic of Korea; ^6^ BK21 FOUR KNU Creative BioResearch Group Kyungpook National University Daegu Republic of Korea

**Keywords:** cell therapy, cerebellar ataxia, intrathecal transplantation, mesenchymal stem cells, neuroinflammation, neuroprotection, Purkinje cell, spinocerebellar ataxia

## Abstract

**Introduction:**

Cerebellar ataxia (CA) encompasses hereditary and acquired disorders unified by Purkinje cell loss and neuroinflammation, for which no disease‐modifying therapy exists. Human mesenchymal stem cells (hMSCs) offer multimodal neuroprotection through paracrine secretion of neurotrophic factors and immunomodulatory mediators.

**Methods:**

We reviewed preclinical and clinical evidence for hMSC therapy across multiple CA etiologies, integrating findings from neuroinflammatory, toxic/developmental, and genetic mouse models alongside published clinical trials and case reports. A systematic literature search was conducted in PubMed/MEDLINE, Embase, and the Cochrane Library (search period: 2000–2026) using the following key terms: “mesenchymal stem cell” AND “cerebellar ataxia”; “MSC” AND “spinocerebellar ataxia”; “hMSC” AND “Purkinje cell”; “stem cell therapy” AND “ataxia”. Inclusion criteria encompassed: peer‐reviewed original research articles and reviews in English; in vivo animal model studies; clinical trials, case series, and case reports. Studies addressing non‐CA neurological conditions without CA‐relevant data were excluded.

**Results:**

hMSC transplantation consistently improved motor function, preserved Purkinje cell integrity, and attenuated neuroinflammation across LPS‐induced, Ara‐C‐induced, and SCA2 transgenic models. A critical observation is that MSCs from CA patients exhibit markedly reduced anti‐inflammatory secretome capacity compared with healthy‐donor MSCs, justifying an allogeneic strategy. Therapeutic efficacy was maintained even after symptom onset in the SCA2 model. A published case report demonstrated safety and preliminary functional benefit of intrathecal allogeneic bone marrow‐derived MSCs in a sporadic adult‐onset ataxia patient.

**Conclusions:**

hMSC therapy targets convergent CA pathomechanisms—microglial suppression, neurotrophin restoration, and Purkinje cell preservation—through a paracrine rather than cell‐replacement mechanism. Post‐symptomatic efficacy and an emerging clinical evidence base support advancing toward placebo‐controlled randomized trials.

## Graphical abstract

Allogeneic hMSCs transplanted across LPS, Ara‐C, and SCA2 cerebellar ataxia (CA) models suppress neuroinflammation and restore the neurotrophin axis, collectively preserving Purkinje cell integrity. These preclinical findings are being translated clinically, from a first‐in‐human case report to an ongoing Phase II/III randomized trial (NCT02540655, NCT06397274), aiming to evaluate motor recovery, Purkinje preservation, and disease modification in CA patients.

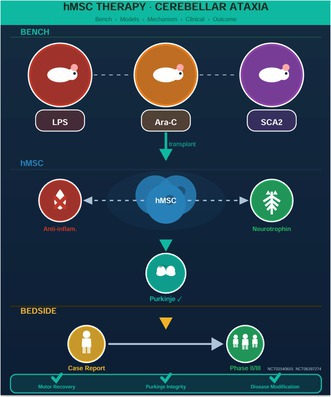

## 1 Introduction

Cerebellar ataxia (CA) comprises a clinically and genetically heterogeneous group of neurological disorders unified by progressive cerebellar degeneration. Cardinal features—gait instability, appendicular dysmetria, dysarthria, and oculomotor abnormalities—arise from Purkinje cell loss and disruption of cerebellar circuitry regardless of the underlying cause [1, 2]. Hereditary forms include more than 50 spinocerebellar ataxia (SCA) subtypes, Friedreich's ataxia (FRDA), and ataxia‐telangiectasia (A‐T); acquired forms encompass immune‐mediated, toxic/drug‐induced, and sporadic adult‐onset ataxia (SAOA) [3]. Global prevalence is estimated at 26 per 100,000, yet no disease‐modifying therapy has been approved for any CA subtype [4].

Despite mechanistic diversity, CA subtypes share convergent pathological substrates: microglial activation with pro‐inflammatory cytokine release (TNF‐α, IL‐1β, IL‐6), deficient neurotrophic support (BDNF, GDNF, IGF‐1), and Purkinje cell apoptosis [5, 6]. This convergence provides the conceptual basis for therapeutic strategies that target downstream pathological mechanisms rather than individual upstream genetic defects.

Human MSCs satisfy ISCT criteria—plastic adherence, CD73/CD90/CD105 positivity, CD45/CD34/CD11b negativity, and trilineage differentiation capacity—and are distinguished by their paracrine immunomodulatory and neurotrophic properties [7, 8]. Upon transplantation, hMSCs secrete BDNF, GDNF, IGF‐1, PGE2, IDO, and TSG‐6, collectively attenuating neuroinflammation and supporting neuronal survival [9, 10]. Practical advantages—ready isolation from bone marrow or umbilical cord, low immunogenicity enabling allogeneic use, and scalable banking—make hMSCs a clinically tractable platform [8].

This review appraises the preclinical and clinical evidence for hMSC therapy in CA, delineates the mechanistic framework emerging from multiple research groups, and positions these findings within the broader landscape of stem cell strategies for ataxia, including neural stem cell (NSC) approaches in SCAs, hematopoietic stem cell transplantation (HSCT) in A‐T, and gene‐edited HSCT in FRDA. A translational roadmap for future clinical development is proposed.

Unlike prior reviews that address stem cell therapy in neurodegenerative diseases broadly, the present review is distinguished by three features: (i) systematic integration of evidence from multiple original studies spanning CA etiologies under a unified paracrine mechanistic framework; (ii) the discovery that CA patient‐derived MSCs exhibit an intrinsic secretome deficiency—directly informing the choice of allogeneic donor strategy; and (iii) post‐symptomatic efficacy data representing the most clinically realistic preclinical design to date, providing direct rationale for enrolling symptomatic patients in future trials.

## 2 Shared Pathomechanisms and Animal Models

Neuroinflammation is a central driver of CA progression across etiologies. Activated microglia and reactive astrocytes release TNF‐α, IL‐1β, and IL‐6 in quantities sufficient to trigger Purkinje cell apoptosis via TNF receptor‐1 and IL‐1 receptor‐mediated caspase activation [11]. In polyQ SCAs, soluble mutant ataxin fragments activate pattern‐recognition receptors on microglia, establishing a feedforward inflammatory cascade [12]. Concurrently, neurotrophic factor depletion—particularly BDNF and GDNF—compromises Purkinje cell survival signaling through TrkB and GFRα1/RET pathways [13]. These two interacting axes, neuroinflammation and neurotrophic deprivation, represent the principal therapeutic targets addressable by hMSC paracrine activity.

A lipopolysaccharide (LPS) intracerebellar injection model reproduces the neuroinflammatory CA phenotype—dose‐dependent Purkinje cell loss, microglial activation, and TNF‐α/IL‐1β elevation—and was validated as a reproducible platform by Hong et al. [14]. Neonatal cytarabine (Ara‐C) administration produces cerebellar granule cell hypoplasia modeling chemotherapy‐induced developmental CA [15, 16]. The ATXN2 transgenic (Tg) mouse, expressing mutant human ataxin‐2 under the Pcp2 Purkinje cell promoter, recapitulates progressive SCA2 with motor symptom onset at 20–24 weeks, enabling post‐symptomatic treatment paradigms that mirror clinical reality [17, 18]. Together, these three models span neuroinflammatory, structural‐toxic, and genetic etiologies—the principal CA categories encountered clinically.

The selection of these specific models was deliberate: the LPS model offers maximal reproducibility and is applicable to cause‐unspecific acute neuroinflammatory CA; the Ara‐C model recapitulates chemotherapy‐induced developmental cerebellar toxicity, a clinically important acquired CA phenotype; and the SCA2 ATXN2 transgenic mouse models one of the most prevalent autosomal dominant SCAs with a progressive natural history amenable to post‐symptomatic treatment paradigms. We acknowledge that immune‐mediated CA (e.g., anti‐GAD65 antibody‐associated) and Friedreich's ataxia models were not included, representing a limitation of the current evidence base that future studies should address.

## 3 MSC Biology and Therapeutic Rationale

The dominant mechanism of hMSC neuroprotection is paracrine secretion rather than direct neuronal differentiation, which occurs at negligible rates [19]. hMSC‐derived PGE2 binds EP2/EP4 receptors on microglia, activating cAMP‐PKA signaling and suppressing NF‐κB‐mediated TNF‐α and IL‐12 production while upregulating IL‐10 [10]. TSG‐6 independently attenuates microglial NF‐κB activation through a CD44‐dependent mechanism [20]. Together, these mediators shift the cerebellar microglial population from pro‐inflammatory M1‐like to anti‐inflammatory M2‐like phenotype: although the M1/M2 microglial phenotype represents a classical framework, it remains widely employed for convenience despite the increasing recognition of microglial heterogeneity [21]. In parallel, hMSC‐secreted BDNF and GDNF activate pro‐survival PI3K‐Akt and MAPK/ERK signaling in Purkinje cells, while IGF‐1 supports granule cell viability [13]. hMSC‐derived extracellular vesicles (EVs) extend this paracrine influence by transferring microRNAs and proteins that modulate neuronal gene expression [22].

A critical observation, reported by Kim et al. [23], is that MSCs isolated from CA patients exhibit markedly reduced secretion of follistatin‐like 1 (FSTL1), transforming growth factor‐beta 1 (TGFB1), insulin‐like growth factor‐binding protein 3 (IGFBP3), and growth arrest‐specific 6 (GAS6), compared with age‐matched healthy‐donor MSCs, despite meeting standard ISCT identity criteria. This secretome impairment mirrors reports of compromised MSC function in other neurodegenerative contexts [24, 25] and indicates that the CA disease process itself modifies MSC paracrine capacity. The mechanistic basis for this impairment likely involves epigenetic reprogramming of MSCs in the context of chronic cerebellar neuroinflammation and oxidative stress: accumulation of reactive oxygen species and sustained pro‐inflammatory cytokine signaling (TNF‐α, IL‐1β) in the CA milieu may induce DNA methylation changes and histone modifications at promoter regions of FSTL1, TGFB1, and IGFBP3, thereby suppressing their transcription. Analogous disease‐induced MSC secretome impairment has been reported in amyotrophic lateral sclerosis [25] and Parkinson's disease models [24], suggesting that disease‐associated stem cell dysfunction is a broadly relevant biological principle rather than a CA‐specific phenomenon. The finding provides direct mechanistic justification for an allogeneic healthy‐donor strategy—the approach adopted in subsequent clinical translation—and suggests that potency assays targeting anti‐inflammatory secretome components should be incorporated into donor qualification protocols.

## 4 Preclinical Evidence Across CA Models

### 4.1 Neuroinflammatory Model

Nam et al. [26] transplanted allogeneic healthy‐donor hMSCs into the LPS‐induced CA model established by Hong et al. [14] (Table 1). hMSC‐treated mice showed significantly improved rotarod performance and normalized motor coordination relative to vehicle‐treated controls, with benefit sustained over the four‐week observation period. Histologically, Purkinje cell loss was reduced from approximately 40% to 15% (calbindin‐D28K immunostaining). Cerebellar TNF‐α and IL‐1β expression was markedly suppressed, and the proportion of morphologically activated Iba‐1+ microglia was significantly reduced, consistent with PGE2‐ and TSG‐6‐mediated M1‐to‐M2‐like microglial reprogramming [10]. These findings align with earlier reports of MSC‐mediated neuroinflammation suppression in experimental autoimmune encephalomyelitis model [27] and spinal cord injury patients [28], confirming that anti‐inflammatory paracrine activity is a reproducible MSC therapeutic mechanism across CNS inflammatory conditions.

**TABLE 1:** Integrated comparison of preclinical hMSC studies across representative CA models.

Parameter	LPS model	Ara‐C model	SCA2 model	Significance
Publication	Nam et al. [26]	Park et al. [15]	Kim et al. [17]	Three independent efficacy studies
CA Etiology	Acquired/Neuroinflammatory	Acquired/Toxic‐Developmental	Hereditary/Genetic (polyQ SCA2)	Broadest etiological coverage
Treatment Timing	Therapeutic (3 days post‐LPS)	Established deficit (10–22 week post‐Ara‐C)	Post‐symptomatic (after confirmed onset)	Most clinically realistic design
Motor Outcome	↑ Rotarod (~80% recovery), simple composite phenotype scoring system, consisting of ledge test and hindlimb clasping test	↑ Rotarod (~50% recovery), simple composite phenotype scoring system Open‐field locomotion	↑ Rotarod (~95% recovery) Slowed progression Preserved function	Consistent benefit across designs
Purkinje Cell Protection	Loss: 40% → 15% (calbindin‐D28K)	Density maintained Cerebellar weight improved	Preserved Histology confirmed	Neuroprotection, not cell replacement
Key Mechanism	Microglia M1 → M2 TNF‐α/IL‐1β ↓ (complete inhibition) TSG‐6 ↑ (~2 fold increase)	BDNF/GDNF ↑ (1.1 ~ 2.4 fold increase) Host‐tissue induction Paracrine crosstalk	Host‐MSC crosstalk Endogenous neurotrophin ↑ (1.2 ~ 2.0 fold increase)	Paracrine‐dominant; etiology‐agnostic

Abbreviations: Ara‐C, cytarabine; BDNF, brain‐derived neurotrophic factor; CA, cerebellar ataxia; GDNF, glial cell line‐derived neurotrophic factor; LPS, lipopolysaccharide; SCA2, spinocerebellar ataxia type 2; TNF‐α, tumor necrosis factor alpha; TSG‐6, tumor necrosis factor‐stimulated gene 6.

### 4.2 Toxic/Developmental Model

Park et al. evaluated hMSC transplantation in adult mice subjected to neonatal Ara‐C exposure [15] (Table 1). Treatment significantly improved rotarod and open‐field locomotion scores over a 12‐week follow‐up. Cerebellar granule cells and Purkinje cells were better preserved, and cerebellar weight was improved. A mechanistically notable finding was that BDNF and GDNF upregulation occurred in hMSCs‐engrafted cerebellum, indicating paracrine induction of neurotrophic factor expression in host tissue—a “host tissue reprogramming” effect that may sustain neuroprotection beyond the lifespan of engrafted cells. Similar paracrine‐mediated neurotrophic induction has been described in MSC studies in other CNS conditions, suggesting a shared mechanism [29].

### 4.3 Genetic SCA2 Model—Post‐Symptomatic Efficacy

Kim et al. administered hMSCs to ATXN2 Tg mice only after confirmed motor symptom onset, recapitulating the clinical scenario in which patients seek treatment after diagnosis [17] (Table 1). Despite post‐symptomatic initiation, rotarod performance decline was significantly attenuated: treated mice retained approximately 70% of baseline motor function versus 35%–40% in vehicle controls at 50 weeks. Purkinje cell density was better preserved—suggesting that hMSC efficacy operates downstream of the primary protein aggregation pathology through a disease‐agnostic neuroprotective mechanism. Host‐MSC crosstalk‐driven BDNF and GDNF induction in surviving Purkinje cells was identified as the primary effector mechanism, corroborating the host tissue reprogramming concept. Post‐symptomatic efficacy has also been reported for MSC therapy in SCA3/MJD models, where repeated systemic MSC administration in Tg‐ATXN3‐69Q mice with established motor deficits produced sustained improvements in rotarod performance and Purkinje cell preservation, reinforcing the generalizability of this finding [30]. Chang et al. further demonstrated that systemic hMSC delivery in SCA2 Tg mice preserved Purkinje cells, indicating that route of administration represents an important optimization variable [31].

## 5 Clinical Evidence and Translation

### 5.1 Published Case Report

Ko et al. reported the first clinical application of allogeneic bone marrow‐derived MSC (BM‐MSC) therapy in a 60‐year‐old male with progressive SAOA (SARA score 14/40 at baseline) [32]. Allogeneic BM‐MSCs from a healthy pre‐screened donor—qualified by secretome potency assays informed by the Kim et al. finding [23]—were delivered intrathecally in three cycles at four‐month intervals (3 × 10^7^ cells per infusion). No serious adverse events were recorded; mild transient CSF pleocytosis resolved within 7 days. SARA score improved to 10/40 at 6 months (a four‐point reduction exceeding the estimated minimally important clinical difference of 2.4 points) [33], with sustained improvement at 12 months (SARA 12/40). Cerebellar MRI volumetry showed no further atrophy over the observation period. This case constitutes proof‐of‐concept for safety and feasibility of intrathecal allogeneic BM‐MSC therapy in CA. However, as a single‐patient uncontrolled report, it cannot exclude placebo effects, natural disease fluctuation, or observer bias; the current clinical evidence is therefore explicitly limited to safety and feasibility proof‐of‐concept. Efficacy conclusions require placebo‐controlled randomized evidence, as discussed in Section 8.

### 5.2 Broader Clinical Trial Landscape

The most advanced MSC product in clinical development for SCA is Stemchymal (Steminent Biotherapeutics Inc., Taiwan), an allogeneic adipose‐derived MSC therapy administered intravenously as an off‐the‐shelf product. Beyond the conventional paracrine neuroprotection mechanism shared with bone marrow‐derived hMSCs, Stemchymal exhibits a distinctive additional mechanism: secretion of a specific paracrine factor that induces cellular autophagy, thereby facilitating degradation of mutant Ataxin‐3—the polyglutamine‐expanded protein responsible for SCA3 pathology (NCT02540655; NCT06397274). This autophagy‐inducing mechanism, supported by preclinical SCA3 transgenic mouse data demonstrating significant clearance of mutant Ataxin‐3 and motor function improvement, is protected by US patent and positions Stemchymal as a potential first‐in‐class candidate for polyglutamine disorders.

In April 2025, Steminent presented full Phase II results at the World Orphan Drug Congress (WODC) USA in Boston (April 2025). The completed Taiwan trial (NCT02540655) was a randomized, double‐blind, placebo‐controlled design in which patients with moderate‐to‐severe SCA3 (SARA ≥ 9) received three intravenous doses of Stemchymal over 12 months. Statistically significant disease stabilization was observed on both the SARA and fSARA (functional SARA) scales, with the treatment group achieving approximately 1.3 points improvement in fSARA score at 12 months—a clinically meaningful contrast to the 1.41–1.6‐point annual SARA worsening reported in untreated SCA3 natural history cohorts from the United States and Europe. Some patients exhibited more than one‐point improvement in SARA score. A parallel Phase II multi‐center trial in Japan, encompassing both SCA3 and SCA6 patients, confirmed consistent positive outcomes across a different ethnic population, reinforcing the cross‐ethnic generalizability of the findings.

Regarding the regulatory and global development trajectory: Steminent completed its Common Technical Document (CTD) submission package for Japan in June 2025 and commenced pre‐submission meetings with its Japanese licensing partner REPROCELL Inc. in preparation for a conditional marketing application. Conditional marketing approval in Taiwan is similarly targeted. In the United States, a Phase IIb trial has been registered with ClinicalTrials.gov (NCT06397274; sponsor: Steminent US Inc.; design: randomized, double‐blind, placebo‐controlled, parallel‐group; intervention: intravenous Stemchymal vs. placebo; target population: polyglutamine SCA). These data collectively establish Stemchymal as the most clinically advanced MSC product in the SCA field and represent a pivotal “bedside” milestone that directly validates the translational potential of MSC‐based therapy for hereditary cerebellar ataxia.

Phase I/IIa evidence has also been reported in broader SCA populations. Tsai et al. [34] conducted an open‐label Phase I/IIa study in Taiwan enrolling 6 patients with SCA3 and 1 with MSA‐C, demonstrating that a single intravenous infusion of allogeneic adipose‐derived MSCs was well tolerated over 12 months, with disease progression appearing to stabilize in the majority of SCA3 patients relative to the expected natural history of 3‐point annual SARA worsening. Jin et al. [35] reported functional improvements following intrathecal umbilical cord MSC infusion in 16 genomically‐confirmed SCA patients, with improvements documented on the International Cooperative Ataxia Rating Scale (ICARS) and Berg Balance Scale (BBS). The favorable safety profile across MSC sources and routes was corroborated by other studies [36]. A systematic review and meta‐analysis by Bhartiya et al. [37] pooling 3 studies (*n* = 47) found a non‐significant trend toward improvement in ICARS/BBS scores and concluded that MSC therapy appears safe but that the absence of randomized controlled trials (RCT) level evidence precludes definitive efficacy conclusions. A study by Appelt et al. [38] supported no statistically significant functional improvement across pooled studies. Critically, no study has reported MSC‐related serious adverse events including ectopic tissue formation or malignant transformation, establishing a consistent safety foundation for advancing to RCTs.

## 6 Integrated Mechanistic Framework

**FIGURE 1:**
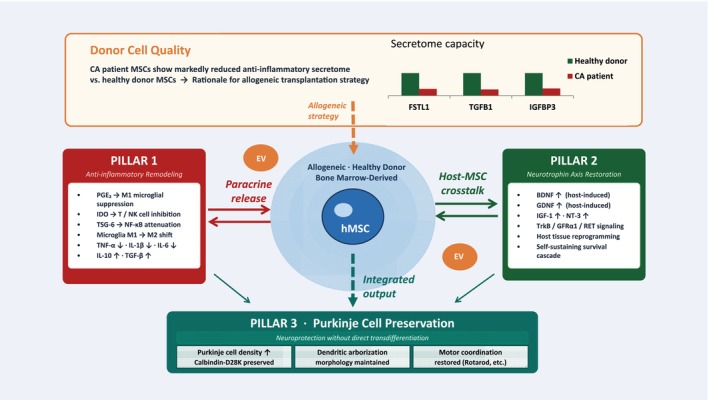
Unified mechanistic framework of human mesenchymal stem cell (hMSC) therapy in cerebellar ataxia. *Upper panel—Donor Cell Quality*. Bar graphs display normalized secretome capacity for three paracrine mediators (FSTL1, TGFB1, and IGFBP3) in mesenchymal stem cells (MSCs) isolated from cerebellar ataxia (CA) patients versus healthy allogeneic donors. CA patient‐derived MSCs exhibit markedly reduced secretome output relative to healthy donor MSCs across all three proteins, providing the mechanistic rationale for an allogeneic rather than autologous transplantation strategy. The orange dashed downward arrow (Allogeneic strategy) indicates the donor selection approach adopted in subsequent preclinical and clinical investigations. *Central panel—hMSC and paracrine network*. The central diagram represents an allogeneic, healthy donor bone marrow‐derived hMSC surrounded by a halo depicting the sphere of paracrine influence within the cerebellar microenvironment. An orange oval badge (EV) denotes extracellular vesicles as secondary mediators of cell‐to‐cell signaling. Red bidirectional arrows labeled “Paracrine release” indicate secretion from hMSC toward host cerebellar tissue; green bidirectional arrows labeled “Host‐MSC crosstalk” indicate reciprocal signaling from host tissue back to the transplanted cells. The dashed teal vertical arrow (“Integrated output”) represents the convergent downstream effect of both paracrine axes onto Purkinje cell survival. *Pillar 1—Anti‐inflammatory remodeling* (*left*, *red*). hMSC paracrine secretion suppresses neuroinflammation through multiple molecular mechanisms: PGE₂ suppresses M1 microglial activation; IDO inhibits T‐ and NK‐cell effector functions; TSG‐6 attenuates NF‐κB signaling. These mediators collectively drive M1‐to‐M2 microglial polarization and reduce pro‐inflammatory cytokine burden (TNF‐α ↓, IL‐1β ↓, IL‐6 ↓) while upregulating anti‐inflammatory mediators (IL‐10 ↑, TGF‐β ↑). *Pillar 2—Neurotrophin axis restoration* (*right*, *green*). Host‐MSC paracrine crosstalk drives endogenous upregulation of neurotrophic factors within cerebellar tissue: BDNF ↑ and GDNF ↑ (both host‐induced), IGF‐1 ↑, and NT‐3 ↑. Signal transduction proceeds through cognate receptors (TrkB for BDNF; GFRα1/RET for GDNF). This host tissue reprogramming establishes a self‐sustaining neurotrophin survival cascade that operates independently of direct MSC engraftment or transdifferentiation. *Pillar 3—Purkinje cell preservation* (*bottom*, *teal*). The integrated output of Pillars 1 and 2 converges on preservation of Purkinje cell structural and functional integrity across all three CA model systems. Key outcome endpoints include: increased Purkinje cell density confirmed by Calbindin‐D28K^+^ immunostaining; maintained morphology; and restoration of motor coordination. BDNF, brain‐derived neurotrophic factor; BM‐MSC, bone marrow‐derived mesenchymal stem cell; CA, cerebellar ataxia; EV, extracellular vesicle; FSTL1, follistatin‐like protein 1; GDNF, glial cell line‐derived neurotrophic factor; GFRα1, GDNF family receptor alpha‐1; hMSC, human mesenchymal stem cell; IDO, indoleamine 2,3‐dioxygenase; IGF‐1, insulin‐like growth factor‐1; IGFBP3, insulin‐like growth factor‐binding protein 3; IL‐1β, interleukin‐1 beta; IL‐6, interleukin‐6; IL‐10, interleukin‐10; M1/M2, pro‐/anti‐inflammatory microglial phenotypes; MSC, mesenchymal stem cell; NF‐κB, nuclear factor kappa‐light‐chain‐enhancer of activated B cells; NT‐3, neurotrophin‐3; PGE2, prostaglandin E2; RET, rearranged during transfection (receptor tyrosine kinase); SCA2, spinocerebellar ataxia type 2; TGF‐β, transforming growth factor‐beta; TGFB1, transforming growth factor beta‐1 (protein); TNF‐α, tumor necrosis factor‐alpha; TrkB, tropomyosin receptor kinase B; TSG‐6, TNF‐stimulated gene 6 protein.

The data from multiple research groups converge on three mechanistic pillars (Figure 1).

Pillar 1—Anti‐inflammatory remodeling. hMSC‐secreted PGE2 and TSG‐6 reprogram cerebellar microglia from M1 to M2‐like phenotype, reducing TNF‐α, IL‐1β, and IL‐6 production [10, 26]. This was most directly demonstrated in the LPS model [26], where Iba‐1+ microglial density and activation morphology were markedly suppressed, but it is also evident in the reduced secondary neuroinflammation observed in the SCA2 model [17]. IDO‐mediated T‐cell suppression provides an additional immunomodulatory layer [39].

Pillar 2—Neurotrophin axis restoration. Host‐MSC paracrine crosstalk induces BDNF and GDNF upregulation in surviving host Purkinje cells and granule neurons, not only in transplanted cells [15, 17]. This “host tissue reprogramming” may sustain neuroprotective benefit beyond the window of transplanted cell survival and represents a therapeutically significant amplification mechanism. The importance of neurotrophic support for Purkinje cell viability is well established [13], and neurotrophic factor delivery has been a consistent goal across multiple CA therapeutic approaches.

Pillar 3—Purkinje cell preservation. Across all three models, Purkinje cell density and morphological integrity were significantly better maintained in hMSC‐treated animals without evidence of MSC‐to‐neuron transdifferentiation. This observation is consistent with the paracrine‐dominant model and indicates that hMSC‐mediated neuroprotection is disease‐agnostic—effective regardless of whether the initiating insult is inflammatory, toxic, or genetic. The CA patient MSC secretome deficiency [23] connects all three pillars: only healthy‐donor MSCs can deliver the full complement of anti‐inflammatory and neurotrophic paracrine signals required for optimal Pillar 1 and 2 activity, thereby explaining why the allogeneic strategy is mechanistically superior.

## 7 Broader Landscape of Stem Cell Therapy in Ataxia

Neural stem/progenitor cell (NSC/NPC) transplantation offers a mechanistically distinct approach to SCA: structural neuronal replacement alongside bystander neuroprotection. Oliveira Miranda et al. demonstrated that murine NSC transplantation into SCA3/MJD mice preserved Purkinje cells, elevated cerebellar neurotrophins, reduced neuroinflammation, and improved motor coordination, with donor‐derived cells expressing mature neuronal markers [30, 38]. Mendonca et al. advanced this with human iPSC‐derived neuroepithelial stem cells (NESCs) in MJD mice, documenting graft survival at 6 months [40]. Notably, MJD patient‐derived NESCs exhibited reduced BDNF‐related gene expression compared with healthy‐donor NESCs [40]—an observation that parallels the CA patient MSC secretome impairment reported by Kim et al. [23], suggesting that disease‐associated stem cell dysfunction is a broadly relevant biological principle. Despite structural replacement potential, NSC approaches face greater manufacturing complexity, immunogenicity, and tumor formation risk than hMSC therapy.

In A‐T, HSCT corrects the hematopoietic immunodeficiency—restoring naïve CD4^+^/CD8^+^ T‐cell populations and reducing lymphoma risk—but does not reverse neurological progression [41]. A recent systematic review confirmed that HSCT shows potential for immune reconstitution in A‐T but has minimal effect on neurological decline and is not currently recommended as standard treatment [42]. Residual ATM‐deficient neurons continue to degenerate despite successful hematopoietic reconstitution, illustrating the principle that cell therapy must target the pathological compartment driving neurological morbidity. hMSC‐mediated paracrine neuroprotection could theoretically complement HSCT as a CNS‐directed adjunct in A‐T.

In FRDA, CRISPR‐Cas9‐corrected autologous HSPCs—in which the GAA repeat expansion is deleted to restore frataxin expression—are advancing toward first‐in‐human application [43]. Omaveloxolone (Skyclarys), the first FDA‐approved drug for FRDA, provides partial symptomatic benefit, underscoring the unmet need for regenerative strategies [44]. In contrast to paracrine hMSC therapy, gene‐corrected HSCT targets the root frataxin deficiency and is therefore the mechanistically appropriate primary strategy for FRDA. iPSC‐derived MSCs from healthy donors could further address the manufacturing and secretome consistency limitations of primary BM‐MSC isolation, potentially overcoming the patient‐derived cell dysfunction documented in CA [23, 45].

## 8 Challenges and Future Directions

Several risks, limitations, and challenges of hMSC therapy warrant explicit acknowledgment to provide a balanced perspective. First, immunogenicity: although allogeneic MSCs exhibit immune privilege through low MHC‐II expression and active immunosuppressive secretion, repeated intrathecal infusions carry a theoretical risk of allosensitization; immune monitoring—including donor‐specific antibody titers and T‐cell alloresponse assays—should be incorporated into future trial protocols. Second, MSC source variability: secretome composition differs substantially across bone marrow‐, adipose‐, and umbilical cord‐derived MSCs, and even within the same tissue source across donors; standardized release criteria based on functional secretome potency assays are therefore essential. Third, optimal dosing strategy—cell number per infusion, interval between cycles, and total number of infusions—remains empirically undefined for CA; dose‐ranging as a primary objective of Phase I/IIa trials is therefore critical. Fourth, long‐term safety data beyond 12 months are absent for CA; systematic surveillance for ectopic tissue formation, malignant transformation, and neurological deterioration must be built into trial design.

**FIGURE 2:**
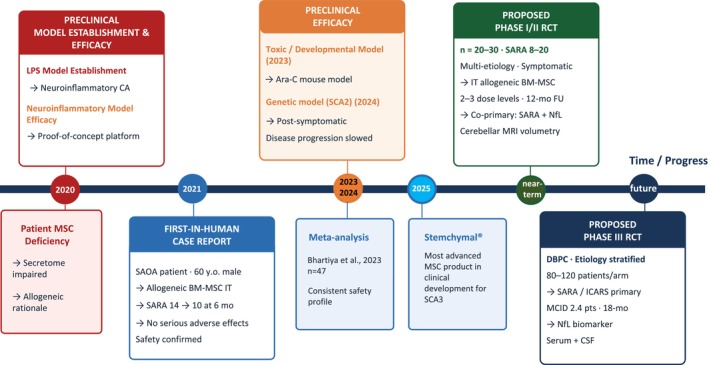
Translational roadmap from hMSC bench research to clinical Phase III in cerebellar ataxia. The figure presents a simplified translational timeline (horizontal axis: Time/Progress) proceeding from preclinical research through first‐in‐human experience to proposed randomized clinical trials. Timeline nodes are labeled with the corresponding year or projected timeframe. *Node 1* (*2020*)*—Preclinical model establishment and efficacy*. Upper card: Hong et al. [14] established a lipopolysaccharide (LPS)‐induced neuroinflammatory CA mouse model as a reproducible preclinical platform (“Proof‐of‐concept platform”), and Nam et al. [26] demonstrated hMSC therapeutic efficacy in this model (“Neuroinflammatory Model Efficacy”). Lower card: Kim et al. [23] demonstrated markedly impaired secretome capacity (FSTL1, TGFB1, IGFBP3) in CA patient‐derived MSCs compared to healthy donor MSCs (“Patient MSC Deficiency”), establishing the scientific rationale for allogeneic donor transplantation. *Node 2* (*2021*)*—First‐in‐human case report*. Ko et al. [32] reported intrathecal allogeneic BM‐MSC transplantation in a 60‐year‐old male patient with sporadic adult‐onset ataxia (SAOA). The SARA score improved from 14 to 10 at 6‐month follow‐up with no serious adverse effects, establishing proof‐of‐concept clinical safety. *Node 3a* (*2023–2024*)*—Extended preclinical efficacy and meta‐analytic evidence*. Upper card: Park et al. [15] extended hMSC efficacy to the Ara‐C toxic/developmental CA model; Kim et al. [17] demonstrated disease‐modifying effects in SCA2 ATXN2 transgenic mice following post‐symptomatic hMSC administration, providing direct preclinical support for treating already‐symptomatic patients. Lower card: A meta‐analysis by Bhartiya et al. [37] (*n* = 47) confirmed a consistent safety profile of MSC therapy across published clinical studies. *Node 3b* (*2025*)*—Stemchymal Phase II randomized double‐blind placebo‐controlled trial*. The first controlled trial to report statistically significant SARA score improvement in SCA3 patients compared to placebo—characterizing it explicitly as a landmark “bedside” milestone (see the text for details in Section 5.2, etc.). *Node 4a* (*near‐term*)*—Proposed Phase I/II RCT*. A multi‐etiology, symptomatic CA cohort (*n* = 20–30; baseline SARA 8–20) receiving intrathecal (IT) allogeneic BM‐MSC at 2–3 dose levels with 12‐month follow‐up. Co‐primary endpoints: Change in SARA score and serum neurofilament light chain (NfL) concentration. Cerebellar MRI volumetry is included as an exploratory structural endpoint. *Node 4b* (*future*)*—Proposed Phase III RCT*. Double‐blind, placebo‐controlled RCT (DBPC) stratified by CA etiology subtype (80–120 patients per arm; SARA and ICARS as co‐primary endpoints; minimal clinically important difference [MCID] 2.4 points; 18‐month follow‐up). NfL from both serum and cerebrospinal fluid (CSF) is incorporated as an objective neurodegeneration biomarker. ATXN2, ataxin‐2; BM‐MSC, bone marrow‐derived mesenchymal stem cell; CA, cerebellar ataxia; CSF, cerebrospinal fluid; DBPC, double‐blind, placebo‐controlled; FU, follow‐up; hMSC, human mesenchymal stem cell; ICARS, International Cooperative Ataxia Rating Scale; IT, intrathecal; LPS, lipopolysaccharide; MCID, minimal clinically important difference; MRI, magnetic resonance imaging; MSC, mesenchymal stem cell; NfL, neurofilament light chain; RCT, randomized controlled trial; SARA, Scale for the Assessment and Rating of Ataxia; SAOA, sporadic adult‐onset ataxia; SCA, spinocerebellar ataxia.

Key scientific challenges include defining the optimal cell dose, infusion frequency, and delivery route for cerebellar targeting. Intrathecal delivery achieves higher posterior fossa cell concentrations than intravenous administration and is supported by clinical safety data [34, 35], but comparative pharmacokinetic studies remain lacking. Cell survival following transplantation is transient—preclinical tracking studies indicate that allogeneic MSCs administered intrathecally are typically detectable for 1–4 weeks before declining to undetectable levels, with few or no cells persisting beyond 4–8 weeks in the CNS environment [9, 20]. This limited engraftment window appears sufficient to initiate paracrine‐mediated host tissue reprogramming, consistent with a “hit‐and‐run” therapeutic paradigm in which brief secretome exposure triggers self‐sustaining neuroprotective cascades. Clearance of allogeneic MSCs involves multiple concurrent mechanisms: early apoptosis driven by the hypoxic and nutrient‐limited CNS microenvironment [46]; progressive immune‐mediated elimination by host CD8^+^ T cells and natural killer (NK) cells as the initial immunosuppressive secretome wanes; and phagocytosis of apoptotic debris by resident microglia and infiltrating macrophages [8]. Importantly, these clearance mechanisms do not appear to generate a pro‐inflammatory secondary response in the preclinical CA models reviewed herein, supporting the safety of the transient engraftment strategy. Regarding long‐term safety, available clinical data—including the 12‐month follow‐up reported by Ko et al. [32] and the open‐label series of Tsai et al. [34]—have not identified immune‐mediated rejection, delayed hypersensitivity, or ectopic tissue formation attributable to allogeneic MSC administration. However, these observation periods are insufficient to exclude late immune sensitization following repeated infusion cycles, which represents an important surveillance priority. The theoretical risk of allosensitization is mitigated by the transient nature of MSC engraftment and by MSC‐intrinsic immunosuppressive mechanisms—including PGE_2_‐ and IDO‐mediated T‐cell suppression—that attenuate the host alloresponse during the therapeutic window; nevertheless, systematic monitoring of donor‐specific antibody titers and T‐cell alloresponse beyond 12 months should be incorporated as a mandatory safety endpoint in Phase II/III protocols. Yet the host tissue reprogramming mechanism [15, 17] suggests that brief paracrine signaling may initiate self‐sustaining neuroprotective cascades; biomaterial encapsulation to extend MSC viability represents a potential engineering solution [47]. Long‐term safety surveillance beyond 12 months is needed (Figure 2).

MSC‐derived extracellular vesicles (MSC‐EVs) warrant specific attention as a next‐generation formulation. Compared to live MSCs, MSC‐EVs offer several CMC advantages: they can be lyophilized for long‐term storage without cryopreservation of living cells; their smaller size (~100–300 nm) may enable superior blood–brain barrier (BBB) penetration; they carry no risk of ectopic tissue formation or malignant transformation; and their cargo (miRNAs, proteins, lipids) can be characterized and standardized more precisely than whole‐cell secretomes. However, MSC‐EVs currently lack the adaptive, context‐responsive secretion capacity of live MSCs—which can dynamically modulate their output in response to host tissue signals—and remain at the preclinical stage for CA. The MSC secretome data from Kim et al. [23] directly informs the selection of EV cargo targets: loading EVs with recombinant FSTL1, TGFB1, or IGFBP3 could represent a rational engineering strategy to maximize therapeutic potency.

To overcome patient MSC secretome impairment [23], several strategies are under investigation: ex vivo inflammatory priming (IFN‐γ or hypoxic conditioning) to upregulate IDO and PGE2 expression [19]; genetic engineering to overexpress BDNF or GDNF; and MSC‐derived EV/exosome preparations as cell‐free alternatives that retain paracrine cargo while avoiding engraftment risks [22, 48].

Chemistry, manufacturing, and controls (CMC) represent a critical but under‐addressed translational bottleneck for hMSC therapy in CA. Potency assay standardization is a regulatory prerequisite: based on the secretome deficiency identified by Kim et al. [23], release criteria for allogeneic MSC products should include quantitative thresholds for FSTL1, TGFB1, and IGFBP3 secretion, alongside standard viability, sterility, and identity criteria; FSTL1 in particular represents a candidate potency biomarker given its direct anti‐inflammatory and neuroprotective activity. Manufacturing scale‐up from conventional 2D monolayer culture to 3D bioreactor systems (stirred‐tank or hollow‐fiber bioreactors) is essential for ensuring batch‐to‐batch consistency and meeting the cell numbers required for multi‐patient trials; 3D bioreactor expansion has also been shown to enhance MSC paracrine activity relative to 2D culture. Implementation of automated closed‐system manufacturing platforms reduces contamination risk, eliminates operator‐dependent variability, and substantially lowers per‐dose production costs—all prerequisites for eventual commercial‐scale supply of an allogeneic off‐the‐shelf MSC product for CA.

The clinical evidence base requires progression from open‐label series to placebo‐controlled RCTs. A Phase I/IIa study (*n* = 20–30; SARA 8–20; 12‐month follow‐up) should evaluate safety, dose‐finding, and pharmacodynamic biomarkers including CSF BDNF/GDNF and serum neurofilament light chain (NfL)—a validated neurodegeneration biomarker [49]. A Phase III RCT should be double‐blind, placebo‐controlled, and stratified by CA etiology (hereditary vs. acquired; SCA genotype where applicable; baseline SARA severity tertile; disease duration), powered for the SARA minimally important clinical difference of 2.4 points [33] over 18 months in 80–120 patients per arm. Secondary endpoints should include ICARS, patient‐reported quality of life (EQ‐5D‐5L), and MRI cerebellar volumetry. Delivery route—intrathecal versus intravenous—should be formally compared in a Phase I/IIa sub‐study before Phase III route selection. Infusion frequency (single vs. repeat cycles at 3‐month intervals) should likewise be assessed as a dosing variable. The post‐symptomatic efficacy data from Kim et al. [17] directly supports enrollment of symptomatic patients without requiring the ethical and operational challenges of pre‐symptomatic detection [50].

The registration of the Stemchymal Phase IIb trial in the United States (ClinicalTrials.gov NCT06397274) provides a directly applicable design precedent for future BM‐hMSC trials in hereditary SCA: randomized, double‐blind, placebo‐controlled, parallel‐group allocation, intravenous or intrathecal delivery, with SARA and fSARA as co‐primary endpoints. Adopting a harmonized endpoint structure across MSC product trials—irrespective of cell source—would facilitate cross‐trial comparisons and accelerate regulatory review.

## 9 Conclusion

Evidence from multiple research groups across neuroinflammatory, toxic/developmental, and genetic CA models establishes a convergent mechanistic case for hMSC therapy: paracrine anti‐inflammatory remodeling and neurotrophin axis restoration collectively preserve Purkinje cells through a disease‐agnostic mechanism. The finding that CA patient‐derived MSCs have impaired secretome capacity provides a mechanistic rationale for healthy‐donor allogeneic transplantation. Post‐symptomatic efficacy in the SCA2 model—the most clinically realistic preclinical design—and a published case report demonstrating intrathecal safety and preliminary benefit represent the current translational frontier. Important limitations must be acknowledged: the clinical evidence base currently comprises a single uncontrolled case report and efficacy conclusions cannot be drawn without placebo‐controlled data; the animal models employed do not fully recapitulate human CA heterogeneity; and key translational variables including optimal dose, infusion schedule, and delivery route remain empirically undefined. Within the broader ataxia cell therapy landscape, hMSC therapy occupies a distinct niche: the most extensively characterized, multi‐etiology applicable, and clinically most advanced paracrine neuroprotection platform. The Phase II results of Stemchymal—demonstrating statistically significant SARA/fSARA stabilization in SCA3 patients versus placebo across independent Taiwan and Japan cohorts, with a US Phase IIb trial registered (NCT06397274) and regulatory submissions underway in Japan and Taiwan—constitute the strongest available controlled clinical evidence for MSC therapy in hereditary ataxia and directly validate the translational niche occupied by allogeneic MSC therapy in this field. Placebo‐controlled, biomarker‐guided Phase II/III trials, incorporating CA‐subtype stratification, CMC‐standardized allogeneic MSC products, and EV‐based next‐generation formulations, are the necessary next step toward clinical practice.

## Funding

This work was supported by the Basic Science Research Program of the Korea National Research Foundation (Ministry of Science, ICT and Future Planning; No. RS‐2024–00408736, RS‐2026–25469872).

## Conflicts of Interest

The authors declare no conflicts of interest.

## Data Availability

The data that support the findings of this study are available on request from the corresponding author. The data are not publicly available due to privacy or ethical restrictions.
